# A universal speed limit for spreading of coherence

**DOI:** 10.1038/s41586-025-09735-z

**Published:** 2025-11-12

**Authors:** Gevorg Martirosyan, Martin Gazo, Jiří Etrych, Simon M. Fischer, Sebastian J. Morris, Christopher J. Ho, Christoph Eigen, Zoran Hadzibabic

**Affiliations:** Cavendish Laboratory, University of Cambridge, Cambridge, UK

**Keywords:** Ultracold gases, Statistical physics, Quantum physics, Bose-Einstein condensates

## Abstract

Discoveries of fundamental limits for the rates of physical processes, from the speed of light to the Lieb–Robinson bound for information propagation^1,2^, often lead to breakthroughs in the understanding of the underlying physics. Here we observe such a limit for a paradigmatic many-body phenomenon, the spreading of coherence during the formation of a weakly interacting Bose–Einstein condensate^3–18^. We study condensate formation in an isolated homogeneous atomic gas^19,20^ that is initially far from equilibrium, in an incoherent low-energy state, and condenses as it relaxes towards equilibrium. Tuning the interatomic interactions that drive condensation, we show that the spreading of coherence through the system is initially slower for weaker interactions and faster for stronger ones, but always eventually reaches the same limit, at which the square of the coherence length grows at a universal rate given by the ratio of Planck’s constant and the particle mass, or, equivalently, by the quantum of velocity circulation associated with a quantum vortex. These observations are robust to changes in the initial state, the gas density, and the system size. Our results provide benchmarks for theories of universality far from equilibrium^21–34^, are relevant for quantum technologies that rely on large-scale coherence, and invite similar measurements in other systems.

## Abstract (web summary)

Experiments reveal a speed limit for the spreading of spatial coherence during the formation of a weakly interacting Bose–Einstein condensate.

## Main

Understanding the dynamics of far-from-equilibrium many-body systems, including the emergence of long-range order in such systems, is an outstanding problem in physics, relevant from subnuclear to cosmological length scales^21–34^. Conceptually, far-from-equilibrium relaxation and emergence of coherence have long been linked to decaying turbulence^12,21,23,27^, which often features self-similar scaling dynamics. More recently, within the framework of nonthermal fixed points (NTFPs)^28^, theorists have drawn analogies between such dynamics and the equilibrium properties of systems close to a continuous phase transition. Near the transition to an ordered state of matter, such as a superfluid or a ferromagnet, the system is scale-invariant and its salient properties do not depend on the microscopic details^35^. Analogously, in the NTFP theory, far-from-equilibrium systems, including the early universe undergoing reheating^27^, quark–gluon plasma in heavy-ion collisions^36^, quantum magnets^37^, and ultracold atomic gases^38–43^, generically show dynamic (spatiotemporal) scaling, with scaling exponents that could define far-from-equilibrium universality classes. Recently, far-from-equilibrium dynamic scaling was observed in several experiments with ultracold atoms, in both isolated (relaxing)^44–50^ and continuously driven^51,52^ systems.

Here we go beyond the elegant scaling properties of far-from-equilibrium relaxation^44–50^ to address the crucial question of how long it takes to establish long-range order. We study this problem for the paradigmatic macroscopically coherent state, the weakly interacting Bose–Einstein condensate, which is also a textbook example of a superfluid.

**Fig. 1:**
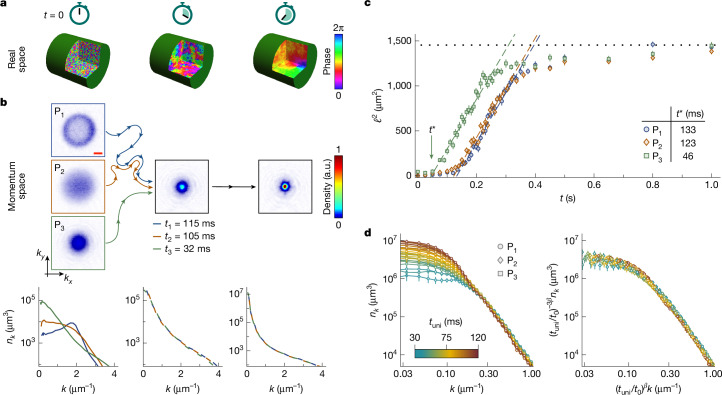
Universal coarsening of an isolated Bose gas. **a**, Real-space cartoon of coarsening. **b**, Momentum-space relaxation for different far-from-equilibrium initial states. Our initial states P_1,2,3_ (left column) have different momentum distributions *n*_*k*_ but the same energy, so the gas always relaxes towards the same equilibrium state. For P_1,2,3_, the system takes different times, *t*_1,2,3_, to evolve to the same *n*_*k*_ shown in the middle column, but from this point onwards, it always evolves in the same way. The *n*_*k*_ distributions are averages of at least 20 measurements. The red scale bar (top image) denotes 1 μm^−1^. **c**, Growth of the coherence length, *ℓ* (see text). Plotting *ℓ*^2^(*t*) reveals three stages of relaxation: (1) the non-universal initial dynamics; (2) the scaling regime in which *ℓ*^2^ grows linearly (dashed lines), as expected for the scaling exponent *β* = 1/2; and (3) the breakdown of scaling at long times owing to finite-size effects. The curves for P_1,2,3_ are parallel, with the initial-state effects captured by the different time offsets t* (intercepts of the dashed lines). **d**, Dynamic scaling. In the scaling regime, the full low-*k* distributions for all three initial states (left panel) can be collapsed onto the same curve (right panel) according to equations (2) and (3) with *β* = 1/2 and *t* → *t*_uni_ ≡ *t* − t*; *t*_0_ = 60 ms is an arbitrary reference time. All error bars show standard errors of the measurements. a.u., arbitrary units.

We study condensate formation in an isolated homogeneous Bose gas^53^, trapped in a cylindrical optical box^19,20^, such as sketched in Fig. 1a (Methods). The gas is prepared far from equilibrium and is initially incoherent but has very low energy and condenses as it relaxes towards equilibrium under the influence of interatomic interactions, characterized by the *s*-wave scattering length *a*. As illustrated in Fig. 1a, in a homogeneous system, the (global) condensate grows through coarsening^25^, the local spreading of coherence. This coarsening is quantified by the growth of the coherence length *ℓ*, over which the first-order correlation function *g*_1_(*r*) decays, and corresponds to narrowing of the momentum distribution *n*_*k*_(*k*) (in which *k* is the wavenumber), which is related to *g*_1_(*r*) by the Fourier transform.

**Fig. 2:**
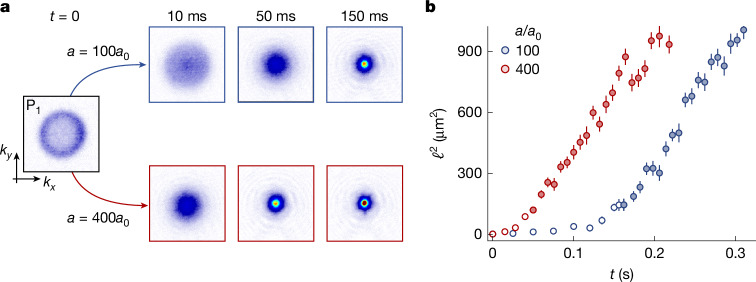
Spreading of coherence for different interaction strengths. **a**, Gas evolution for two different scattering lengths *a*, starting in the same initial state P_1_. For stronger interactions, the condensate (the peak at *k* = 0) emerges sooner. **b**, However, plotting *ℓ*^2^(*t*) reveals that the interaction strength affects only the non-universal initial dynamics (open symbols), whereas the linear growth of *ℓ*^2^ in the universal coarsening regime (solid symbols) is the same for both *a* values. Panels in **a** and data points in **b** show averages based on at least 20 measurements. All error bars show standard errors of the measurements.

**Extended Data Fig. 1:**
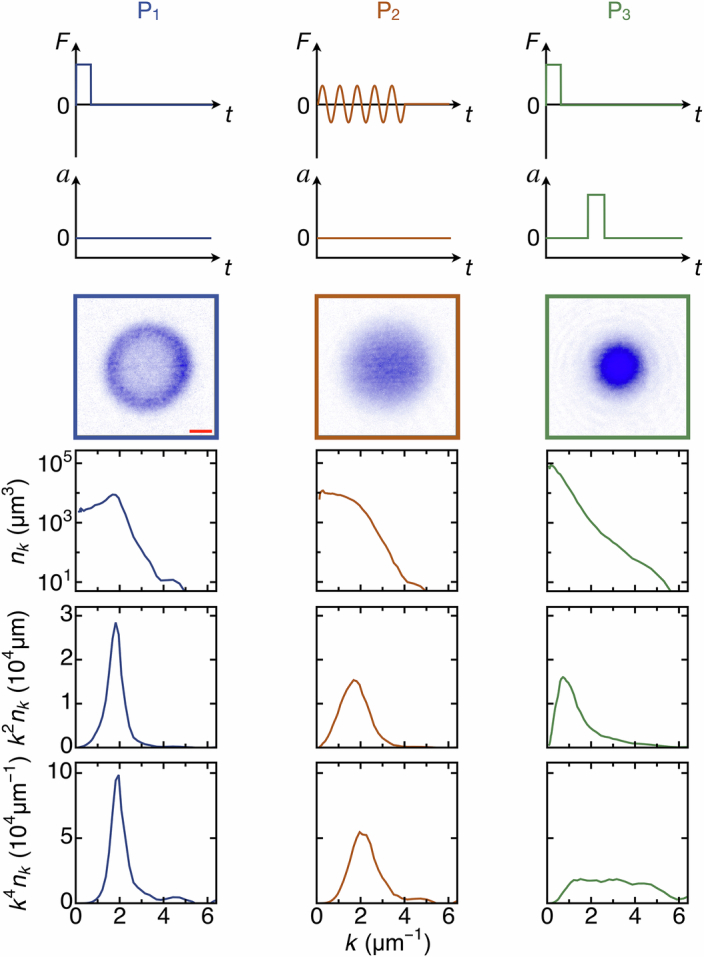
Initial-state preparation. We prepare our initial states using a combination of time-dependent force *F* and interaction pulsing, as shown in the top two rows. The forcing pulses for P_1_ and P_3_ are 8 ms long and have strength *F*_0_ = *k*_B_ × 1.5 nK μm^−1^. The sinusoidal force for P_2_ has amplitude *F*_0_ = *k*_B_ × 0.3 nK μm^−1^ and angular frequency *ω* = 2π × 10 Hz and is applied for 1 s. For P_3_, the interactions are pulsed to *a* = 400*a*_0_ for 30 ms. After the end of the preparation sequence, we wait for 500 ms at *a* = 0 for *n*_*k*_ to become isotropic. The images (same as in Fig. 1b) show the *k*_*x*_–*k*_*y*_ distributions for *k*_*z*_ ≈ 0 just before we turn on interactions to initiate the relaxation; the red scale bar in the left image denotes 1 μm^−1^. The bottom three rows show different moments of the corresponding momentum distributions. The integrals of *k*^2^*n*_*k*_ and *k*^4^*n*_*k*_ are, respectively, proportional to the total atom number and energy, which are the same for all three states; the energy per particle is *ε* = *k*_B_ × 20(2) nK.

**Extended Data Fig. 2:**
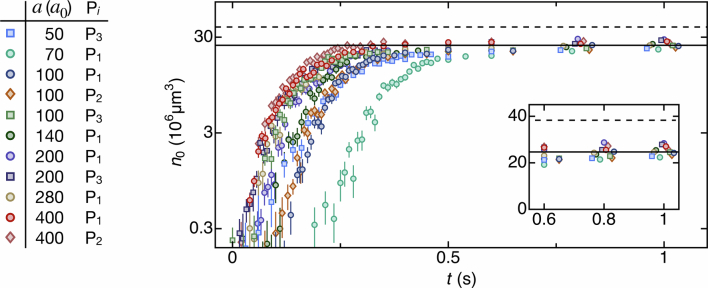
Equilibrium *n*_0_ and *η.* We plot the observed *n*_0_(*t*) for all 11 of our datasets taken with *V* = 55(12) × 10^3^ μm^3^ and *n* = 5.4(1.2) μm^−3^. The solid line shows the long-time saturation value ${\bar{n}}_{0}=25(2)\times 1{0}^{6}\,{\rm{\mu }}{{\rm{m}}}^{3}$, obtained by averaging all of the measurements for the two longest times (0.8 s and 1.0 s for all datasets; the data points for these two times are slightly offset horizontally for visual clarity). The dashed line shows the value of *n*_0_ observed for a quasi-pure Bose–Einstein condensate, ${n}_{0}^{{\rm{BEC}}}=39(2)\times 1{0}^{6}\,{\rm{\mu }}{{\rm{m}}}^{3}$, which gives ${\bar{n}}_{0}/{n}_{0}^{{\rm{BEC}}}=0.64(6)$. The inset shows the same data on a linear scale, focusing on long times.

Our experiments are performed with ^39^K atoms and we tune *a* using a Feshbach resonance, exploring coarsening for *a* = (50–400)*a*_0_, in which *a*_0_ is the Bohr radius. Our cylindrical box has radius *R* = 21(2) μm, length *L* = 40(4) μm, and volume *V* = 55(12) × 10^3^ μm^3^. Our gas density, *n* = 5.4(1.2) μm^−3^, corresponds (assuming ideal-gas thermodynamics) to the critical temperature for condensation *T*_c_ = 127(19) nK. The kinetic energy per particle in our initial incoherent states is *ε* = *k*_B_ × 20(2) nK (in which *k*_B_ is the Boltzmann constant), corresponding to a large equilibrium condensed fraction *η* = 0.61(4) (see Extended Data Figs. 1 and 2). During coarsening, the total particle number, *N* ≈ 3 × 10^5^, is essentially constant (see Methods) and the gas is always weakly interacting in the sense that *na*^3^ < 10^−4^. We measure *n*_*k*_ by absorption imaging (along the *z* direction) after time-of-flight expansion, performing the inverse Abel transform on the line-of-sight integrated distributions; just for the images shown in Figs. 1b and 2a, we instead image only slices of the cloud^54^ corresponding to *k*_*z*_ ≈ 0 (Methods). We normalize *n*_*k*_ such that ∫*n*_*k*_4π*k*^2^d*k* = *N*.

We first show that, although the short-time relaxation dynamics inevitably depend on the details of the initial state, the long-time relaxation does not (Fig. 1b). For this purpose, we engineer three different far-from-equilibrium states, starting with a quasi-pure condensate and using a time-varying force to perturb the cloud (see Extended Data Fig. 1). Our initial states P_1,2,3_ have different *n*_*k*_ (see left column) but the same *ε*. At time *t* = 0, the gas is non-interacting and we then initiate relaxation by switching *a* to 100*a*_0_. Starting from an initial state, *n*_*k*_(*k*) follows some trajectory (represented by the wavy coloured lines) in the space of functions with the same *N* and *ε*. The middle column shows that, still far from equilibrium, these trajectories converge to the same *n*_*k*_. The time that the gas takes to evolve to this *n*_*k*_ depends on the initial state (*t*_*i*=1,2,3_ for P_*i*=1,2,3_) but further evolution from this *n*_*k*_ is the same for all initial states; note that the state trajectories for P_1_ and P_2_ merge before merging with the P_3_ trajectory, but we just show an *n*_*k*_ for which all three have converged.

The long-time relaxation of a low-energy Bose fluid was theoretically studied in different frameworks. In ref. ^23^, this problem was studied for an incompressible superfluid, for which the spreading of coherence is associated with the decay of a random tangle of quantized vortices (variously known as the Kibble’s vortex tangle^22^, superfluid turbulence^55^ and Vinen turbulence^26,33^) and *ℓ* is set by the typical distance between the vortex lines. For *ℓ* ≫ *ξ*, in which *ξ* is the size of the vortex core, the prediction is that

1 $$\frac{{\rm{d}}{\ell }}{{\rm{d}}t}\propto \frac{{\rm{ln}}(A{\ell }/\xi )}{{\ell }},$$

in which *A* is a dimensionless constant. On the other hand, for coarsening of wave excitations, corresponding to a compressible-fluid flow, approximate kinetic equations give

2 $${\ell }(t)\propto {t}^{\beta },$$

with *β* = 1/2 (refs. ^21,34,39,41^). In our weakly interacting gas, with the vortex-core size set by the healing length $\xi =1/\sqrt{8{\rm{\pi }}na}$, both vortices and waves could play a substantial role. Note, however, that the predictions of equations (1) and (2) differ only in a logarithmic correction. Moreover, our measurements of *n*_*k*_ reveal *ℓ* independently of what type of excitations are dominant and limit its value.

For coarsening fully characterized by the growth of *ℓ* (ref. ^25^), the low-*k* momentum distribution exhibits self-similar evolution:

3 $${n}_{k}(k,t)={{\ell }}^{d}(t)f(k{\ell }(t)),$$

in which *d* = 3 is the system dimensionality and *f* is a dimensionless scaling function. In this case, *ℓ*(*t*) is $\propto {n}_{0}^{1/3}(t)$, in which *n*_0_ ≡ *n*_*k*_ (*k* = 0) and the condensate occupation is *N*_0_ = (2π)^3^*n*_0_/*V*. This scaling does not define the absolute value of *ℓ* and here we define it so that *ℓ*^3^ is equal to the system volume when the gas is in equilibrium; to convert the observed *n*_0_ values to *ℓ*, we also take into account the finite *k*-space resolution of our time-of-flight measurements (Methods). We observe good agreement with equations (2) and (3), without the need to invoke the logarithmic correction in equation (1), but this does not exclude its relevance on much larger length scales; note also that the importance of this correction depends on the unknown value of *A*.

To compare our data to equation (2), we first note that it is formally valid only for *t*, *ℓ* → *∞*. However, one can observe the same scaling for finite *t* and *ℓ* by absorbing all the effects of the non-universal initial dynamics into time offsets such as seen in Fig. 1b, that is, by shifting *t* → *t* − t*, in which t* depends on the initial state^49^ (see also ref. ^56^).

**Extended Data Fig. 3:**
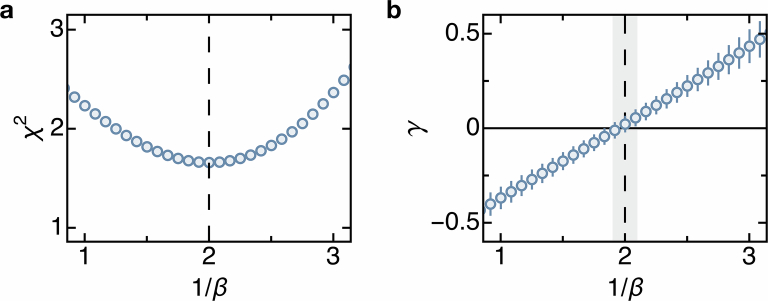
Determining *β.* **a**, The *χ*^2^ values (combined for all 13 datasets summarized in Fig. 3) for the linear long-time fits of *ℓ*^1/*β*^(*t*), showing a minimum at 1/*β* ≈ 2. **b**, The exponent *γ* quantifying the self-consistency of the dependence of d*ℓ*^1/*β*^/d*t* on *na* for various 1/*β* (see text). Self-consistency requires *γ* = 0, which we observe for *β* = 0.50(3) (grey shading).

For *β* = 1/2, in the scaling regime *ℓ* ∝ (*t* − t*)^1/2^ and in Fig. 1c we plot *ℓ*^2^(*t*). This reveals both the scaling regime in which *ℓ*^2^ grows linearly, at a rate that does not depend on the initial state (dashed lines), and the offsets t* for P_1,2,3_; see also Extended Data Fig. 3. As the system approaches equilibrium, for *ℓ*^2^ ≳ 900 μm^2^ ≈ (*V*/2)^2/3^, the scaling breaks down owing to finite-size effects; from here on, we focus on the regime before this breakdown.

**Extended Data Fig. 4:**
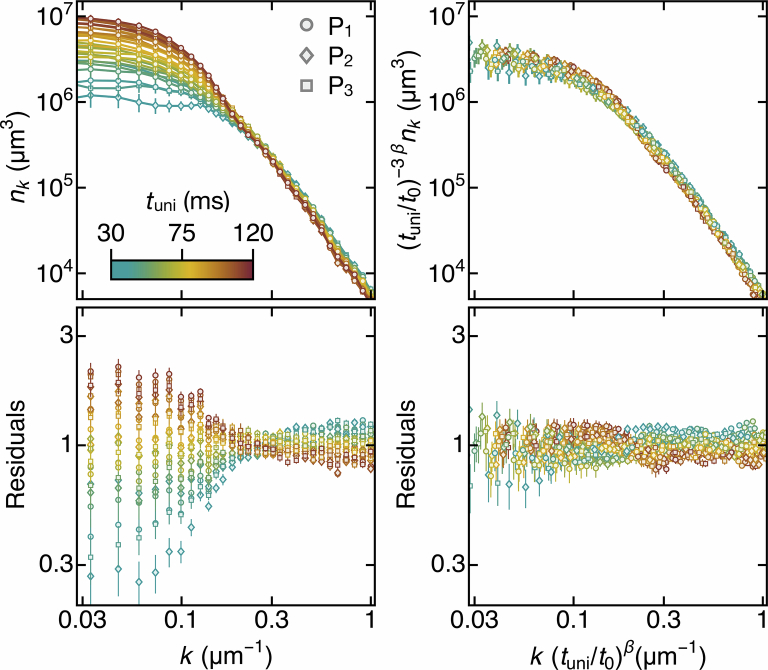
Dynamic scaling residuals. The top panels reproduce Fig. 1d and the bottom panels show the corresponding log-space spread of the data.

In Fig. 1d, using the t* values from Fig. 1c, we show that, in the scaling regime, the low-*k* momentum distributions for all three initial states can indeed be collapsed onto the same universal curve according to equation (3) with *ℓ* ∝ (*t* − t*)^1/2^ (see also Extended Data Fig. 4).

We now turn to varying the strength of the interactions that drive the coarsening and study how this affects the coarsening rate. For *ℓ* ∝ (*t* − t*)^*β*^, we define the ‘speed of coarsening’ as *D* ≡ d*ℓ*^1/*β*^/d*t*, which is (for any *β*) time-invariant in the scaling regime and does not depend on the non-universal t*. For a low-energy Bose gas described by the Gross–Pitaevskii equation, the interactions-set units of length and time are *ξ* and *t*_*ξ*_ ≡ *mξ*^2^/*ħ*, in which *ħ* is the reduced Planck’s constant and *m* the atom mass. Hence, on dimensional grounds, *ℓ*/*ξ* ∝ ((*t* − t*)/*t*_*ξ*_)^*β*^ and we get

4 $$D\propto \frac{{\xi }^{1/\beta }}{{t}_{\xi }}\propto \frac{{\hbar }}{m}{(na)}^{1-1/(2\beta )}.$$

Specifically for *β* = 1/2, as observed in Fig. 1, this has a counter-intuitive implication that *D* = d*ℓ*^2^/d*t* ∝ *ħ*/*m* does not depend on the interaction strength *na* (see also refs. ^21,24^). In the hydrodynamic theory of ref. ^23^, the same result emerges if we neglect the logarithmic correction in equation (1) (which depends on the interactions through *ξ*), because then d*ℓ*^2^/d*t* can only be set by the quantum of velocity circulation associated with a quantum vortex, *κ* = 2π*ħ*/*m*.

In Fig. 2, we show data for coarsening at *a* = 100*a*_0_ and 400*a*_0_, starting from the same initial state P_1_; the 100*a*_0_ data here is the same as in Fig. 1. The images in Fig. 2a show what we intuitively expect—for larger *a*, the condensate emerges sooner. However, in Fig. 2b, plotting *ℓ*^2^(*t*) reveals that the effects of the interaction strength are, just like the initial-state effects, confined to the difference in the non-universal t*—in the scaling regime (solid symbols), the rate of the linear growth of *ℓ*^2^ is essentially the same for both *a*.

**Fig. 3:**
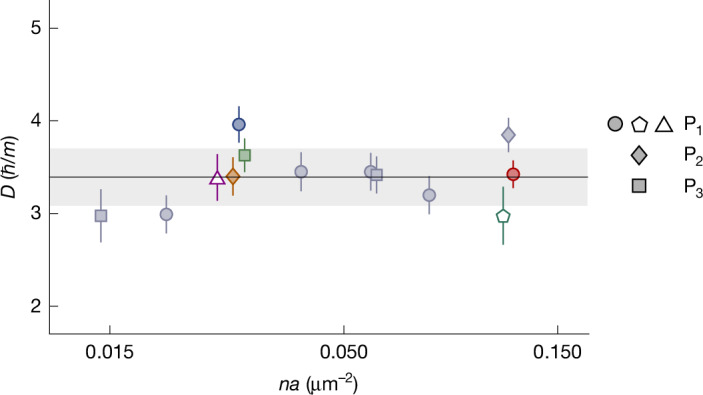
Universality of the coarsening speed *D.* Our measurements for various interaction strengths show no systematic variation of *D* = d*ℓ*^2^/d*t* (in the scaling regime) and give a combined estimate *D* = 3.4(3)*ħ*/*m* (solid line and shading). The four datasets shown in Figs. 1c or 2b are represented here by the corresponding coloured symbols. The purple triangle indicates a measurement for which we reduced the gas density *n* by a factor of 4.2, which reduces *T*_c_ ∝ *n*^2/3^ and the equilibrium condensed fraction (from about 60% to about 30%) but does not affect *D*. The green pentagon indicates a measurement for which we instead reduced the system volume *V* by a factor of 3.5; in this case, *ℓ*^2^ saturates at a correspondingly lower value (∝ *V*^2/3^) but again *D* is not affected. For details on the data taken with reduced *n* or *V*, see Extended Data Fig. 5. The data points corresponding to the same *na* values are slightly offset horizontally for visual clarity. The error bars reflect fitting errors.

**Extended Data Fig. 5:**
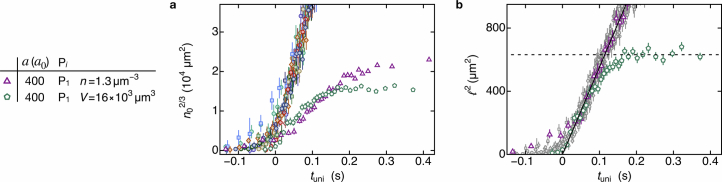
Changing *n* or *V.* **a**, Plotting ${n}_{0}^{2/3}$ versus *t*_uni_ = *t* − t* for all 11 datasets taken with the same *n* and *V* (see Extended Data Fig. 2), together with the two further sets taken with reduced *n* (purple triangles) or *V* (green pentagons), shows that the rate of growth of ${n}_{0}^{2/3}$ depends on the gas density and the system size. **b**, However, the rate of growth of *ℓ*^2^ in the scaling regime is universal. The only effect of the system size is that, for lower *V*, the saturation of *ℓ*^2^ occurs at a lower value (dashed line shows *V*^2/3^ for the smaller box).

We performed such measurements for various scattering lengths and also varied the gas density and system size (see also Extended Data Fig. 5). In each case, we fit the slope *D* = d*ℓ*^2^/d*t* in the scaling regime and summarize our results in Fig. 3. We observe no systematic variation of *D* with the interaction strength *na* and obtain a combined estimate *D* = 3.4(3)*ħ*/*m*; here the error in *D* is purely statistical, whereas the uncertainty in our box volume leads to a systematic error of ±0.5*ħ*/*m* (Methods).

**Fig. 4:**
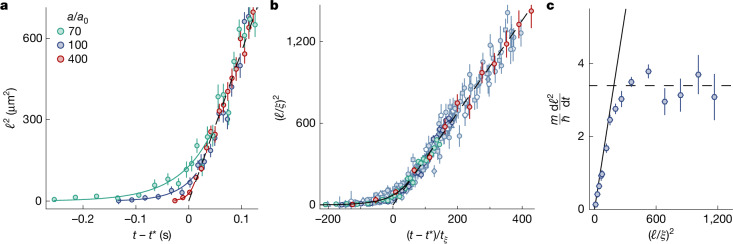
Reaching the coarsening speed limit. **a**, The growth of *ℓ*^2^ for the same initial state (P_1_) and gas density but different *a*. All curves start at *t* = 0 and plotting them versus *t* − t* (with *a*-dependent t*) collapses them in the universal scaling regime. The dashed line shows *ℓ*^2^ = *D*(*t* − t*). For weaker interactions, the system approaches this speed limit slower and reaches it at a larger *ℓ*; the solid lines show exponential fits to the early-time data. **b**, When expressed in the interactions-set units of length, $\xi =1/\sqrt{8{\rm{\pi }}na}$, and time, *t*_*ξ*_ ≡ *mξ*^2^/*ħ*, all of our data for different P_*i*_, *a*, *V*, and *n* (see Fig. 3) collapse onto a single curve, meaning that the speed limit is always reached at the same *ℓ*/*ξ*. The solid line shows exponential growth with a time constant *τ* = 56*t*_*ξ*_ and the dashed line has slope *mD*/*ħ* = 3.4. **c**, Numerically differentiating the data in **b**, we eliminate the non-universal t* and show as a function of (*ℓ*/*ξ*)^2^ how d*ℓ*^2^/d*t* approaches the universal *D* = 3.4*ħ*/*m* and then stops growing; the solid and dashed lines are the same functions as in **b**. For weaker interactions (larger *ξ*), observing this speed limit requires a larger physical system. The data points show averages based on at least 20 measurements. All error bars show standard errors of the measurements.

Finally, in Fig. 4, we study how the system approaches the long-time coarsening speed *D*, which also reconciles our observations with the finite-system intuition (and experience) that, for stronger interactions, condensates form faster, and for *a* → 0, they do not form at all.

As we illustrate in Fig. 4a for fixed *n*, the same initial state (P_1_) and three values of *a*, for weaker interactions the gas takes longer to join the universal scaling trajectory and joins it at a larger value of *ℓ*. Here all curves start at *t* = 0 and plotting versus *t* − t* collapses them in the scaling regime; the dashed line shows *ℓ*^2^ = *D*(*t* − t*).

**Extended Data Fig. 6:**
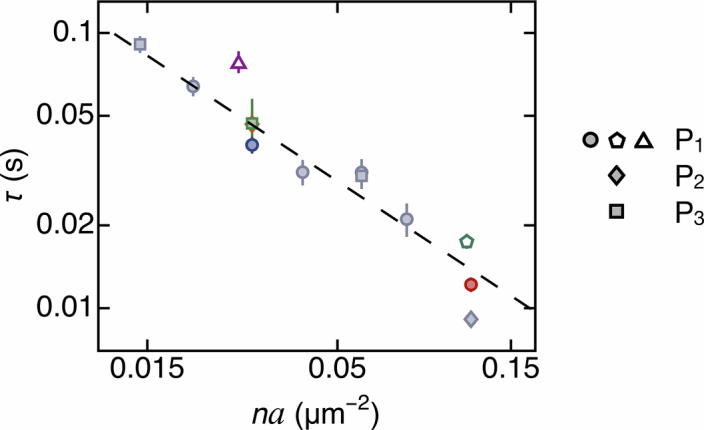
The timescale for the approach to the scaling regime. The timescale *τ* of the exponential fits to the data before the scaling regime, for all 13 datasets summarized in Fig. 3. The dashed line is a power-law fit, giving *τ* ∝ (*na*)^−0.9(1)^, consistent with *τ* ∝ *t*_*ξ*_ ∝ 1/(*na*).

In Fig. 4b, we show that expressing all of our data for different P_*i*_, *a*, *V*, and *n* (see Fig. 3) in terms of *ξ* and *t*_*ξ*_ collapses them onto a single curve. Here the dashed line has slope *mD*/*ħ* = 3.4 and the solid line that captures the approach to the scaling regime is an exponential with a time constant *τ* = 56*t*_*ξ*_ (see also Extended Data Fig. 6). Numerically differentiating these data, and thus eliminating the non-universal t*, in Fig. 4c, we show how d*ℓ*^2^/d*t* approaches the universal *D* = 3.4*ħ*/*m* (dashed line) as a function of (*ℓ*/*ξ*)^2^.

**Extended Data Fig. 7:**
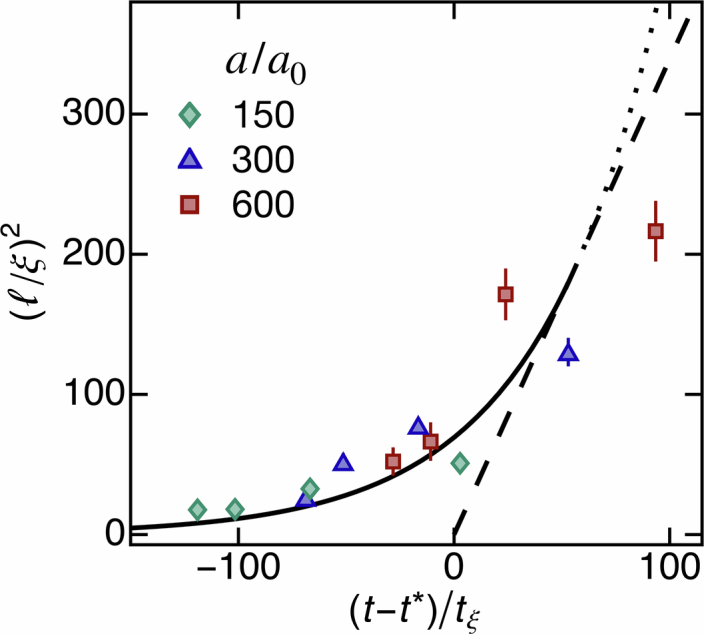
Comparison with the data from ref. ^46^. We present the data corresponding to Fig. 3 in ref. ^46^ in the style of our Fig. 4b and show that they are consistent with our results if we include appropriate time shifts t* (which were not considered in ref. ^46^). The solid and dashed lines are the same as in Fig. 4 and the dotted line is the extension of the former. For the largest *a*, the data reach (*ℓ*/*ξ*)^2^ ≈ 200, at which point they are equally consistent with the speed limit (dashed line) and with the extrapolation of the initial exponential growth (dotted line).

The dimensionless results in Fig. 4b,c imply that, for any interaction strength, the system would eventually, for *ℓ* ≫ *ξ*, exhibit the same coarsening speed *D*. However, the system size required to observe this is larger for larger *ξ* (smaller *na*) and diverges for *na* → 0. As we discuss in Methods and Extended Data Fig. 7, previous experiments on the emergence of extended coherence during far-from-equilibrium condensation, in both harmonic^14^ and box^46^ traps, were not in the universal-speed regime; consequently, for tunable interactions in ref. ^46^, the observed relaxation time was ∝ 1/*a*.

Our results should be relevant across many fields, from cold atoms and the conventional low-temperature physics^26,33^ to cosmology^22,27^ and high-energy physics^40^. The fact that *ħ* and *m* appear in *D* only through their ratio, or the quantum of circulation *κ*, implies that the results are also applicable to systems in which the underlying physics is not quantum^57^. They should also be relevant for benchmarking the theories of ultrarelativistic systems, for which *β* = 1/2 is also predicted^39^, but—in that case—the effective mass, *m*_eff_ ∝ *ħ*/(*ξc*) (in which *c* is the speed of light) and hence *D* ∝*ξc* depend on the interactions.

The value of *D* = 3.4*ħ*/*m*, equal to 5.5 μm^2^ ms^−1^ for ^39^K, has curious implications for the emergence of coherence on truly macroscopic length scales. For example, for coherence to spread by means of coarsening over more than 1 cm would require hours, and even possible logarithmic corrections cannot change this conclusion substantially. However, an interesting question is whether this speed limit can be broken by a fundamentally different preparation protocol, for example, by melting a Mott insulator (through a quantum phase transition) to obtain a superfluid with long-range coherence.

In the future, it would be interesting to disentangle the roles of waves and vortices during coarsening, by directly imaging the latter, to search for possible logarithmic corrections to the coarsening speed, and to perform similar measurements for fermionic superfluids and gases with long-range interactions.

## Methods

### Optical box trap

Our cylindrical optical box is made of 532-nm light and has a trap depth of approximately *k*_B_ × 300 nK. Our standard box of volume *V* = 55(12) × 10^3^ μm^3^, used for most measurements, has radius *R* = 21(2) μm and length *L* = 40(4) μm. For the extra measurement with the smaller *V* = 16(3) × 10^3^ μm^3^, we use a box with *R* = 14(1) μm and *L* = 26(3) μm. Note that, in both cases, *V*^1/3^ ≈ 2*R* ≈ *L*.

### Measuring the momentum distribution *n*_*k*_(*k*)

Our experiments are performed with the lowest hyperfine ground state of ^39^K. We measure *n*_*k*_(*k*) after a time-of-flight expansion (at the start of which we set *a* → 0), first optically pumping the atoms to the highest hyperfine ground state and then imaging them. To deduce *n*_*k*_ values that vary over six orders of magnitude, we combine measurements with time-of-flight duration in the range 16–120 ms; the longest time of flight gives the best *k*-space resolution, relevant for low *k*, whereas shorter ones give better signal-to-noise ratio at large *k*. Even for our longest time of flight, the optical density at *k* ≈ 0 is high for clouds with a high condensed fraction, so to deduce high column densities while keeping optical density ≲ 2, we pump a variable fraction of atoms (down to about 3%) into the imaging state^58^.

For the images shown in Figs. 1b and 2a, and in Extended Data Fig. 1, we image only slices of the cloud^54^ corresponding approximately to *k*_*z*_ ∈ [−0.1, 0.1] μm^−1^, using a thin sheet beam to pump the atoms to the imaging state. Note that the thickness of our sheet pumping beam is not perfectly uniform, resulting in a slight left-to-right asymmetry in the optical density; this is most visible in the image of the P_1_ state, for which the asymmetry is the largest, about 10%.

### Initial-state preparation

To prepare our initial states P_1,2,3_, we start with a weakly interacting quasi-pure condensate with *N* = 3.0(1) × 10^5^ atoms^53^, turn off the interactions (*a* → 0) using the Feshbach resonance at 402.7 G (ref. ^59^) and perturb the cloud using a combination of time-dependent forcing (using a magnetic field gradient) and interaction pulsing, as outlined in Extended Data Fig. 1. In a clean, cylindrically symmetric potential, our forcing would result in anisotropic momentum distributions. However, weak disorder that is naturally present in our trap^52^ couples excitations along different directions, so simply waiting for 500 ms at *a* = 0 always results in states with isotropic but far from equilibrium *n*_*k*_.

The forcing parameters are chosen such that P_1,2,3_ all have the same energy per particle, *ε* = *k*_B_ × 20(2) nK; for our 11 datasets for which *V* and *n* are also the same, this corresponds to the same equilibrium state (see Extended Data Fig. 2).

### Deducing *ℓ* from *n*_0_

In this section, we explain how we deduce *ℓ* from the measured *n*_0_, taking into account the finite *k*-space resolution of our time-of-flight measurements.

In Extended Data Fig. 2, we show our 11 main datasets, taken with *V* = 55(12) × 10^3^ μm^3^ and *n* = 5.4(1.2) μm^−3^. At long times, *n*_0_ always saturates to ${\bar{n}}_{0}=25(2)\times 1{0}^{6}\,{\rm{\mu }}{{\rm{m}}}^{3}$ (solid line), which means that the equilibrium condensed fraction *η* is always (approximately) the same. For comparison, for a quasi-pure equilibrium Bose–Einstein condensate (*η* > 0.9), which was never perturbed in the way shown in Extended Data Fig. 1, we observe ${n}_{0}^{{\rm{BEC}}}=39(2)\times 1{0}^{6}\,{\rm{\mu }}{{\rm{m}}}^{3}$ (dashed line).

The ratio ${\bar{n}}_{0}/{n}_{0}^{{\rm{BEC}}}=0.64(6)$ is consistent with our thermodynamic estimate *η* = 0.61(4) for *ε* = *k*_B_ × 20(2) nK. However, in both cases, the observed equilibrium *n*_0_ is, owing to the finite *k*-space resolution, lower than the theoretical ${n}_{0}^{{\rm{th}}}={N}_{0}V\,/{(2{\rm{\pi }})}^{3}$ by a factor of *ζ* = 1.7(4), with the error in *ζ* dominated by the uncertainty in the box volume; note that *ζ*^1/3^ = 1.2(1) means that the narrowest observed *n*_*k*_ distributions (corresponding to *ℓ*^3^ = *V*) are 20(10)% broader than the Heisenberg limit set by the box size^60^.

To model the effect of the finite *k*-space resolution, we assume that the true width of the momentum distribution (∝ 1/*ℓ*) and the width of the resolution point spread function add in quadrature, so the apparent coherence length *ℓ*′ is related to *ℓ* by

5 $$1/{{{\ell }}^{{\prime} }}^{2}=1/{{\ell }}^{2}+1/{{\ell }}_{0}^{2},$$

in which *ℓ*_0_ is set by our resolution and we use our equilibrium measurements to calibrate ${{\ell }}_{0}={V}^{1/3}\,/\sqrt{{\zeta }^{2/3}-1}=58(10)\,{\rm{\mu }}{\rm{m}}$. Hence, to deduce *ℓ* from the observed *n*_0_, we first calculate ${{\ell }}^{{\prime} }={({n}_{0}/{n}_{0}^{{\rm{eq}}})}^{1/3}{V}^{1/3}$ using ${n}_{0}^{{\rm{eq}}}=\zeta {\bar{n}}_{0}$ and then calculate ${{\ell }}^{2}={{{\ell }}^{{\prime} }}^{2}\,/(1-{({{\ell }}^{{\prime} }/{{\ell }}_{0})}^{2})$. Note that if we numerically model the effect of the finite resolution as a convolution of the true *n*_*k*_ with a Gaussian point spread function of width *σ*_*k*_, our *ζ* = 1.7 corresponds to *σ*_*k*_ ≈ 0.04 μm^−1^.

For the values of *ℓ* reported in the main text, we use the central values for *V* and *ℓ*_0_ and the error in *D* = 3.4(3)*ħ*/*m* is purely statistical, arising from the data scatter. The correlated errors in *V* and *ℓ*_0_ correspond to a systematic error in *D* of ±0.5*ħ*/*m*.

### The scaling exponent *β*

In the main text, we show that our data are consistent with *β* = 1/2. Here we provide further analysis to confirm this.

First, at long times, *ℓ*^1/*β*^(*t*) is ∝ *t* − t* only for the correct *β*. We perform such linear fits for all 13 of our datasets (see Fig. 3) assuming different values of *β* and in Extended Data Fig. 3a show the combined *χ*^2^ values for all 13 fits, which show a minimum at 1/*β* ≈ 2.

Second, ignoring the linear-fit quality for different *β* values, for self-consistency, d*ℓ*^1/*β*^/d*t* should be ∝ (*na*)^1−1/(2*β*)^ (equation (4)), so *C* = (*na*)^1/(2*β*)−1^d*ℓ*^1/*β*^/d*t* should be independent of *na*. Fitting *C* ∝ (*na*)^*γ*^ for all 13 datasets, we obtain *γ*(*β*), shown in Extended Data Fig. 3b. We see that the results are self-consistent (*γ* = 0 within errors) only for *β* = 0.50(3).

### Dynamic scaling of *n*_*k*_

As a complement to Fig. 1d, in Extended Data Fig. 4 (bottom panels), we show the log-space spread of the data around their geometric mean at each *k*, both before (left) and after (right) the dynamic scaling.

For this dynamical collapse, we do not attempt to account for the effects of our finite *k*-space resolution (see Methods section ‘Deducing *ℓ* from *n*_0_’), which would involve numerically deconvolving the observed *n*_*k*_(*k*) distributions with a model point spread function. For these data, *ℓ*/*ℓ*_0_ < 0.5 and most points lie at *k* ≫ *σ*_*k*_, so the resolution effects should be small. Numerically convolving model functions similar to our *n*_*k*_ and a Gaussian with our *σ*_*k*_, we find indeed that the effects on the *n*_*k*_ values are on the order of a few percent, much smaller than the residual spread in the bottom-right panel of Extended Data Fig. 4.

### Changing *n* or *V*

For two of the measurements summarized in Fig. 3, we reduced either *n* or *V* (while keeping *ε* the same). In Extended Data Fig. 5, we show further details of these measurements. Reducing either *n* or *V* reduces the equilibrium value of *n*_0_ and the rate of growth of ${n}_{0}^{2/3}$ in the scaling regime, but the scaling dynamics of *ℓ*^2^ are universal.

### The time constant *τ* for the approach to the scaling regime

In Extended Data Fig. 6, we show the results of independent exponential fits to the early-time data (the approach to the universal scaling regime) for all 13 datasets summarized in Fig. 3. Fitting *τ* ∝ (*na*)^*δ*^ with a free exponent *δ* (dashed line) gives *δ* = −0.9(1), consistent with *τ* ∝ *t*_*ξ*_ ∝ 1/(*na*).

### Particle loss

The one-body particle loss owing to the background gas in the vacuum chamber and off-resonant light scattering is always small (a few percent) during 1 s of gas relaxation (see Extended Data Fig. 2) but the loss owing to three-body recombination, −d*n*/d*t* ∝ *n*^3^*a*^4^, grows with *n* and *a* and limits the range of interaction strengths that we can explore. For our larger *n* = 5.4(1.2) μm^−3^ and largest *a* = 400*a*_0_, the total particle loss over 1 s is slightly more than 10%; for all other measurements, it is much lower.

### Comparison with previous measurements

The early experiments on condensation dynamics were performed with inhomogeneous gases in harmonic traps^11,13–16^, which makes comparison with uniform-system theory difficult. The closest one to examining the uniform-system physics was ref. ^14^, in which the emergence of coherence in a ^87^Rb gas was examined interferometrically and the authors focused on the quasi-homogeneous region near the trap centre. Still, for a quantitative comparison, a problem is that, in a harmonic trap, the central density grows, and hence *ξ* decreases, during condensation. We therefore make only a rough comparison with our results. In ref. ^14^, coherence spread over 8.5 μm in about 350 ms. However, most of the dynamics happened over about 150 ms, which gives an estimate d*ℓ*^2^/d*t* ≈ 0.5 μm^2^ ms^−1^, about five times lower than 3.4*ħ*/*m*_Rb_ ≈ 2.5 μm^2^ ms^−1^.

In ref. ^46^ (by our group), relaxation was studied in a box trap and for tunable interactions. The box size and the range of *a* values were similar to ours but the initial far-from-equilibrium state was prepared by rapid evaporation, removing 77% of the atoms, so the gas density and the observable values of *ℓ*/*ξ* were lower. The data were within errors consistent with dynamic scaling but the deduced *β* ≈ 0.34 was slightly lower than the expected 1/2 and the characteristic relaxation time was ∝ 1/*a*. However, as with all of the NTFP experiments before ref. ^49^, the approximation |t*| ≪ *t* was implicitly assumed. In Extended Data Fig. 7, we show that these measurements can be aligned with our results in Fig. 4b simply by including appropriate time shifts t* and the observed *a* dependence of the relaxation dynamics is explained by the system not having reached the universal speed limit; all of the data are consistent with the initial exponential growth that has a characteristic timescale *τ* = 56*t*_*ξ*_ ∝ 1/(*na*).

## Online content

Any methods, additional references, Nature Portfolio reporting summaries, source data, extended data, supplementary information, acknowledgements, peer review information; details of author contributions and competing interests; and statements of data and code availability are available at 10.1038/s41586-025-09735-z.

## Data Availability

The data that support the findings of this study are available in the Apollo repository (10.17863/CAM.121960). Any further information is available from the corresponding authors on reasonable request.
